# Neurodevelopmental origin and adult neurogenesis of the neuroendocrine hypothalamus

**DOI:** 10.3389/fncel.2014.00440

**Published:** 2015-01-06

**Authors:** Roberto Maggi, Jacopo Zasso, Luciano Conti

**Affiliations:** ^1^Laboratory of Developmental Neuroendocrinology, Department of Pharmacological and Biomolecular Sciences, Università degli Studi di MilanoMilano, Italy; ^2^Interuniversity Centre for the Research on the Molecular Bases of Reproductive Diseases (CIRMAR)Milano, Italy; ^3^Centre for Integrative Biology (CIBIO), Università degli Studi di TrentoPovo, Italy

**Keywords:** hypothalamus, neurogenesis, adult stem cells, tanycytes, neural progenitor cells, neural stem cell (NSC)

## Abstract

The adult hypothalamus regulates many physiological functions and homeostatic loops, including growth, feeding and reproduction. In mammals, the hypothalamus derives from the ventral diencephalon where two distinct ventricular proliferative zones have been described. Although a set of transcription factors regulating the hypothalamic development has been identified, the exact molecular mechanisms that drive the differentiation of hypothalamic neural precursor cells (NPCs) toward specific neuroendocrine neuronal subtypes is yet not fully disclosed. Neurogenesis has been also reported in the adult hypothalamus at the level of specific niches located in the ventrolateral region of ventricle wall, where NPCs have been identified as radial glia-like tanycytes. Here we review the molecular and cellular systems proposed to support the neurogenic potential of developing and adult hypothalamic NPCs. We also report new insights on the mechanisms by which adult hypothalamic neurogenesis modulates key functions of this brain region. Finally, we discuss how environmental factors may modulate the adult hypothalamic neurogenic cascade.

## Introduction

The hypothalamus is a homeostatic regulator of the neuroendocrine system and of behavioral and physiological processes essential for life processes (i.e., thermoregulation, food and water intake, reproduction, circadian rhythms). Most of these functions develop during prenatal life and defects of hypothalamic maturation may lead to a variety of diseases including obesity, infertility, as well as mood disorders. The endocrine hypothalamus is made of multiple nuclei composed of two neurosecretory families of neurons. The magnocellular neurons, located in the paraventricular (PVN) and supraoptic (SON) nuclei, that project to the posterior lobe of the pituitary to release arginine vasopressin (AVP), and oxytocin (OT) and the parvocellular neurons, positioned in the preoptic (PON), PVN, periventricular (PeVN), arcuate (ARC), and ventromedial (VMN) nuclei that project to the median eminence (ME) where they release corticotropin-releasing hormone (CRH), thyrotropin-releasing hormone (TRH), growth hormone-releasing hormone (GHRH), dopamine (DA), gonadotropin-releasing hormone (GnRH) and somatostatin (SST) in the portal vasculature to control the secretory activity of the anterior pituitary (Swanson, 1987). Unveiling the mechanisms controlling hypothalamic development and its adult neurogenic processes may help to identify the nature of hypothalamic dysfunctions and to develop future therapies. Moreover considering the characteristic heterogeneity/complexity of this system, which establishes connections with multiple central nervous system (CNS) regions, the hypothalamus poses a challenging model for understanding neural patterning and differentiation (Markakis, 2002).

## Development of the hypothalamus

The hypothalamus derives from the most rostro-ventral part of the embryonic prosencephalic neuroepithelium. In mouse, its primordium is morphologically evident from approximately embryonic day (E) 9.5. Initial immunohistochemical studies, carried out in rodents and human (Altman and Bayer, 1978, 1986), have shown that the earlier generated neurons will generate the hypothalamic peripheral region (*lateral zone*). Later, a second neurogenic wave will establish the hypothalamic core (*intermediate zone*), while a subsequent neurogenic wave will generate the neurons of the *midline zone*, directly connected with the retina, pituitary and autonomic centers. The introduction of the prosomeric model (Puelles, 2001), linking the morphological development to gene expression patterns, suggested a new picture for hypothalamic development. This model is based on an initial induction and patterning by specific morphogens such as Sonic Hedgehog and Nodal (Chiang et al., 1996; Mathieu et al., 2002), and on the formation of defined subdomains. The anatomical identity of specific neuronal populations in the embryonic and adult hypothalamus has been well characterized in zebrafish (Mueller and Wullimann, 2002), but is still ambiguous in mammals. Genetic inducible fate mapping studies in mouse have shown that the early Shh-expressing progenitors contribute to form neurons and astrocytes in the mammillary and posterior tuberal regions (that includes VMN and ARC) as well as tanycytes, specialized ependymal bipolar cells present in the ME of the 3rd ventricle (Alvarez-Bolado et al., 2012). Progenitors labeled at later stages give rise to neurons and astrocytes of the entire tuberal region, in particular the VMN, but not to any of the mammillary region and ME, indicating that: (i) the classical *transverse* zones (anterior, tuberal and posterior) of the hypothalamus have defined progenitor domains; and (ii) little or no cell mixing occurs between tuberal and anterior hypothalamus. Finally, neurons of the tuberal hypothalamus destined to mediolateral levels are produced progressively, partially diverging from the “three neurogenic waves” model. On the whole, all of these observations indicate that many of the distinguishable hypothalamic components arise from a mosaic of heterogeneous neuroepithelial sites.

## Embryonic hypothalamic NPCs

**Figure 1:**
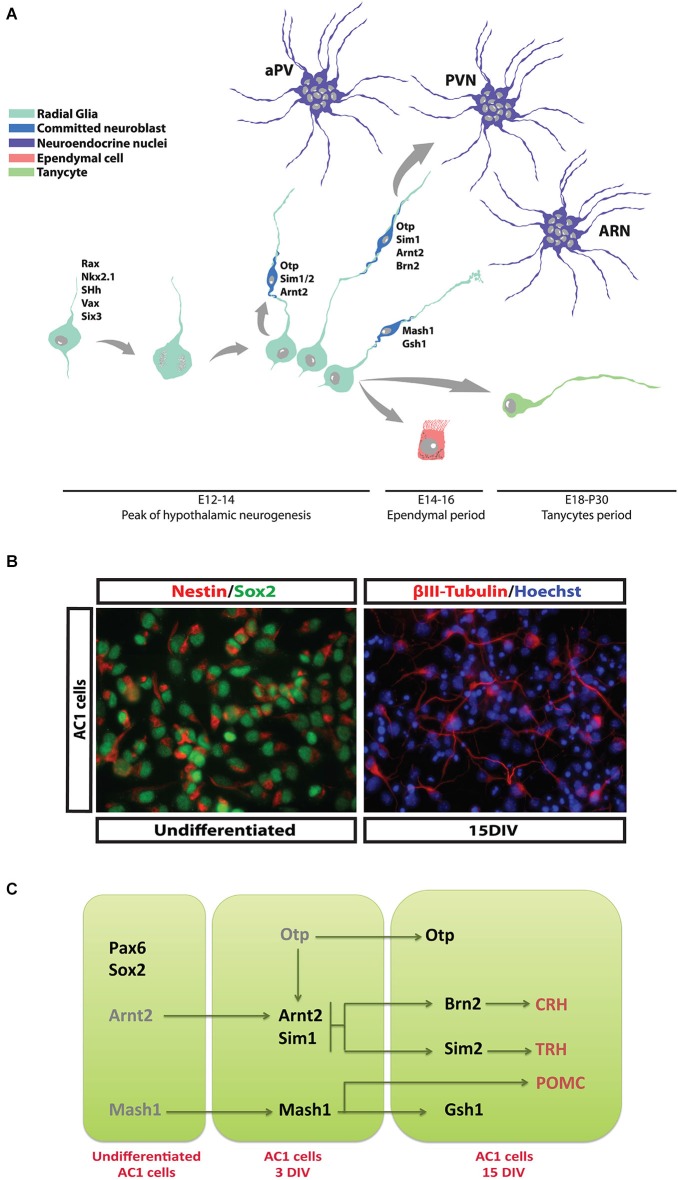
**Differentiation of hypothalamic NPCs during development**. **(A)** At E12.5–14.5 newborn neuroblasts are progressively specified and start the production of specific neurohormones while migrating toward the appropriate hypothalamic nucleus. At later embryonic stage (E16–18) ciliated and non-ciliated ependymal cells are generated and take place along the 3rd ventricle wall. Finally, tanycytes are generated during perinatal period and in the first post-natal days. **(B)** AC1 cells, a NPC population established from fetal hypothalamus. These cells display typical NPC and hypothalamic markers such Sox2 and Nestin respectively (left panel). If prompted to differentiate, they generate mature hypothalamic neurons. **(C)** Schematic diagram reporting the transcription factors expressed by AC1 cells during proliferation and their conversion into specific neurohormone-secreting neuronal subtypes (see Cariboni et al., 2014).

In the developing rat hypothalamus neurogenesis occurs over E10.5–18.5, when new cells are generated by the expansion of NPC pool which delineate two distinct ventricular proliferative zones, the medial part and the ventral part of the 3rd ventricle wall (Sousa-Ferreira et al., 2014; Figure 1A). Evidences suggest that newborn neurons originating from the ventricular proliferative zones migrate by E14.5 toward the mediolateral hypothalamic parenchyma by peripheral apposition with an “outside-in” pattern, so that younger neurons will form, or are incorporated into, the most inner part of the specific nuclei. Neurons migrating laterally will generate the SON, while the ones migrating medially will form the PVN and aPV (Altman and Bayer, 1978; Bayer and Altman, 1987). Various guidance cues, including members of the Netrin, Slit/Robo and Semaphorin/Plexin/Neuropilin families, the Notch effector gene Hes1 and GABA are possibly involved in migration of hypothalamic precursors (Deiner and Sretavan, 1999; Xu and Fan, 2007; Aujla et al., 2011; Stratton et al., 2014). More recently, the chemokine CCL2, a mediator of neuroinflammation, has also been reported to modulate hypothalamic neuronal migration (Poon et al., 2014).

In mouse, the majority of the neuroendocrine neurons become post-mitotic at E12.5–14.5, when they begin to produce specific neurohormones and migrate to their final destinations (Markakis and Swanson, 1997). An important exception is represented by GnRH-producing neurons that derive mainly from the neural precursor cells (NPCs) of the olfactory placode reaching the PON area of the hypothalamus by tangential migration during the embryonic life (Schwanzel-Fukuda and Pfaff, 1990; Cariboni et al., 2007). Other hypothalamic cell populations are generated during the late embryonic period (E16.5–E18.5), including ependymal cells and cuboid ciliated cells that line the 3rd ventricle walls (Rodríguez et al., 2005). Tanycytes represent a defined subgroup of ependymal cells generated between E18.5 and the initial postnatal days by differentiation of a subpopulation of radial glial cells. Their functions are still not completely revealed, but they seem to play a role as adult NPCs (see next sections) and to be involved in feeding behavior and chemoception (Rodríguez et al., 2005; Bolborea and Dale, 2013). A complex genetic program controls the differentiation of hypothalamic NPCs that will form neuroendocrine nuclei; mouse genetic studies have identified a developmental “transcription factors code” characterized by specific temporal and regional patterns of gene expression (Szarek et al., 2010). Along with a series of early patterning genes that determine the development of the whole hypothalamic region (i.e., *Shh, Rax, Nkx2.1, Six3, Vax1*, etc.), *Ortopedia* (*Otp*) has been identified as the master gene regulating the late developmental stages of the NPCs that will give rise to PVN, SON, PeVN and dopaminergic ARC neurons (Acampora et al., 1999; Wang and Lufkin, 2000). Likely, the development of the VMN and the ARC is under the control of proneural genes and transcription factors (*Mash1, Sf-1, Gsh1* and *NHLH2*) (McNay et al., 2006). Other genetic signals (*Arnt2/Sim1)* activated downstream, or sometimes in parallel, to these master genes lead to the specific identity of *Otp* primed hypothalamic NPCs (Keith et al., 2001; Michaud, 2001). Indeed, SIM2 has been involved in the differentiation of NPCs to TRH and SST expressing neurons, while BRN2 in the differentiation and survival of PVN and SON precursors that will express AVP, OT, and CRH (Schonemann et al., 1995).

Although a genetic program has been grossly delineated, the coordination of neuroendocrine NPCs selection and lineage commitment and the specification of defined hypothalamic neuronal identities remain still to be precisely determined. A significant improvement in neurodevelopmental studies has been given by *in vitro* models that allow to recapitulate processes important for the development, like proliferation, migration and differentiation of NPCs (Lein et al., 2005; Polleux and Anton, 2005). The establishment of cultures of hypothalamic neural stem cell (NSC) or precursors is therefore a useful tool to study the selective differentiation of endocrine neurons. The isolation of NPCs with neuroendocrine characteristics from the hypothalamic or other embryonic tissues has been field of intense investigations (Markakis et al., 2004; Wataya et al., 2008; Salvi et al., 2009). Even though each of the so far isolated NPCs show high intrinsic experimental value, their different growth characters (i.e., neurospheres vs. monolayer), low expression of endocrine peptides, anatomical derivation (embryonic stem cells vs. hypothalamic tissue), age of derivation (adult vs. fetal) and low efficiency of neuronal differentiation make these cell preparations of limited application. Recently, we have reported the isolation and characterization of a stable NSC population derived from E13.5 mouse hypothalamus (Cariboni et al., 2014). These cells (named AC1) grow as an adherent culture in defined serum-free medium and express typical markers of neurogenic hypothalamic radial glia (Figure 1B). After prolonged expansion, AC1 cells can be efficiently prompted to differentiate into neurons *in vitro* and start to express some hormonal neuropeptides, like TRH, CRH, and pro-opiomelanocortin (POMC; Figure 1C), suggesting their potentiality to be exploited to elucidate the mechanisms involved in the development of hypothalamic NPCs.

## Neurogenic potential of adult hypothalamic NPCs

**Figure 2:**
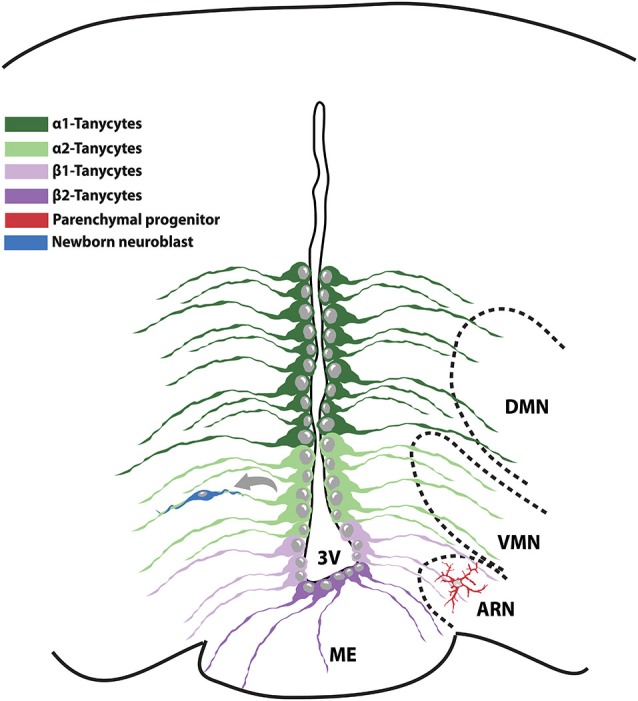
**Schematic representation of the NPC populations existing in the adult hypothalamic niche**. The medial part of the 3V contains α1-tanycytes and proliferative α2-tanycytes that take contact with the VMN and ARN nuclei respectively. β-tanycytes reside on the floor of the 3V and in strict association with the hypothalamic-pituitary portal system. VMN: ventromedial nucleus; ARN: arcuate nucleus; 3V: 3rd ventricle; ME: median eminence.

Since a decade ago, occurrence of neurogenis was thought to be restricted to two defined brain regions, namely the subventricular zone (SVZ) of the lateral ventricle and the subgranular zone (SGZ) of the hippocampal dentate gyrus. Nevertheless, even if at lower rate than in the abovementioned districts, neurogenesis has been described also in the adult hypothalamus (Cheng, 2013). The presence of BrdU-incorporating cells in the rat adult hypothalamus was first reported in 2002 (Evans et al., 2002). Three years later, BrdU-incorporating nestin-positive NPCs were specifically identified in the ependymal layer of the 3rd ventricle of 8-week-old rats (Xu et al., 2005). Some of these NPCs have been classified as tanycytes, a population of postnatal and adult radial glia cells and identified as modulators of neuroendocrine activity and homeostasis (Rodríguez et al., 2005; Lechan and Fekete, 2007; Prevot et al., 2010; Bolborea and Dale, 2013). Differences in gene expression and location in the 3rd ventricle wall have led to the classification of four types of tanycytes. The α1-tanycytes and α2-tanycytes found at the level of VMN and arcuate nuclei (ARN) respectively, with their processes contacting blood vessels and hypothalamic neurons of the adjacent regions. β1-tanycytes, residing in the lateral part of the infundibulum and in contact with GnRH-secreting neurons and endothelial cells. Finally, β2-tanycytes sited on the floor of the 3rd ventricle and in close contact with blood vessels and the hypothalamus-pituitary portal system (Lee and Blackshaw, 2012; Figure 2).

The identification of the exact cell of origin of newborn neurons in the adult hypothalamus is still a debated issue. Indeed, different studies have reported either β-tanycytes or α-tanycytes as the sole hypothalamic NPC population. Nevertheless, it cannot be excluded that, depending on the age, different tanycyte subclasses might act as NPCs (Lee et al., 2012; Haan et al., 2013; Robins et al., 2013). Indeed, while β2-tanycytes have been shown to proliferate and generate a considerable amount of neurons involved in metabolism balance during juvenile period (Lee et al., 2012; Haan et al., 2013), their rate of proliferation substantially declines in adulthood. In this view, β2-tanycytes might represent transient hypothalamic NPCs, later replaced by α2-tanycytes. Also, it can be hypothesized that β-tanycytes and α-tanycytes rather than representing distinct NPC populations, might be one the progenitor of the other. To this regard, Placzek’s group reported that α2-tanycytes proliferate extensively starting from the second month of life and are able to give rise to neurons, astrocytes and other tanycyte subtypes, representing the truly adult hypothalamic NPCs (Robins et al., 2013). In support of this hypothesis, α2-tanycytes have been shown to generate β-tanycytes *in vivo*. Also, it is worth noting that only α2-tanycytes isolated from the adult hypothalamus can generate expandable neurospheres *in vitro*, indicating the unlimited self-renewal ability typical of NPCs. On the contrary, α1-tanycytes and β2-tanycytes exhibit limited and none proliferative capacity, respectively (Robins et al., 2013). Other studies have reported that also the hypothalamic parenchyma harbors potential NPCs (Kokoeva et al., 2007; Li et al., 2012; Haan et al., 2013) thus raising the possibility that a variety of NPC subsets with different function might coexist. To further complicate the scene, a variety of signaling molecules have been linked to modulation of NPCs proliferation by acting in different hypothalamic districts. For instance, BDNF increases cell proliferation in the PVN (Pencea et al., 2001), while fibroblast growth factor-2 (FGF-2) and ciliary neurotrophic factor (CNTF) act within the ARN (Pierce and Xu, 2010).

## Modulation of adult hypothalamic neurogenesis

Damages to homeostatic components, depending on locations in the brain, may lead to impaired neurogenesis, neurodegeneration, cognitive dysfunctions and energy imbalance. The hypothalamus, in particular, controls many functions of the endocrine system and it is itself a target of several circulating hormones that characterize feedback’s control mechanisms. It is also known that during puberty neurogenesis occurs in rodents in a sexually dimorphic pattern in hypothalamic regions that control sexual behavior (Ahmed et al., 2008). Removal of gonadal hormones before puberty eliminates sex differences, indicating that gonadal steroids direct the addition of new cells during puberty to maintain and accentuate sexual dimorphisms in the adult brain. Recently, a sex dimorphism has been reported in the modulation of neurogenesis linked to metabolic modifications (Lee et al., 2014) (see below).

### Metabolic modifications

There are converging line of evidence suggesting that the newly generated hypothalamic neurons may be involved in metabolism, energy balance and weight (Bolborea and Dale, 2013; Lee et al., 2014). Hypothalamic regions identified to be significantly neurogenic, both in basal and stimulated condition, such as the parenchyma, ARC, VMN and DMN, contain neuropeptide Y (NPY) and POMC neurons that are primarily involved in energy balance regulation (Kokoeva et al., 2005). Moreover, some of the adult hypothalamic newborn cells acquire leptin responsiveness, suggesting that they might be integrated in the existing neuronal system to participate in metabolic functioning. Also, it has been reported a sexual dimorphic response of diet-induced modification of neurogenesis in ME. It has been found that in mice maintained on high fat diet (HFD) feeding, neurogenesis in the ME is significantly increased in adult female, but not in male, and it results to be attenuated by prolonged HFD (Lee et al., 2012). This effect may be related to estrogen levels (Bless et al., 2014) and to a different response to the neuroinflammation induced by HFD regimen (Lee et al., 2012). Finally, it has been reported that hypothalamic NSCs derived from obese mice exhibit impaired proliferation and differentiation (Li et al., 2012). The neurogenic NPCs responsive to diet in ME have been identified to be a sub population of tanycytes, likely the β2-tanycytes (Bolborea and Dale, 2013). Actually, the tanycytes have been functionally recognized as glucosensitive cells responsive to metabolic stimulation and signaling changes that are relevant to the control of feeding and energy balance (Lee et al., 2012; Li et al., 2012).

### Neuroinflammation and aging

Neuroinflammation is considered a complex response of CNS to different insults, like toxins, neurodegeneration and metabolic alterations and can be activated by either pathological conditions or excessive neuronal activity.

Due to the diversity of cell types involved and their activation states, neuroinflammation has been described to be either detrimental or supportive for adult neurogenesis (Ekdahl et al., 2009). Several studies have consistently shown that neuroinflammation can be an hallmark of hypothalamic aging and stress response to nutritional excess (Cai, 2013). Indeed, high rate of hypothalamic inflammation and reduced neurogenesis have been observed in mediobasal hypothalamus (MBH) of aged mice. Although acute inflammatory responses are known to provide obligatory defensive mechanisms of the body, persistent neuroinflammation has been identified as a primary assaulting candidate for impaired neurogenesis, NSC survival and differentiation (Ekdahl et al., 2003; Li et al., 2012).

A recent study by Zhang et al. (2013) has proposed a mechanism to elucidate the pivotal hypothalamic role in the program of systemic aging. The authors showed that activation of microglial NFkB and TNF-α release reduce neurogenesis in aged mice by a decline of hypothalamic GnRH levels. Noteworthy, systemic GnRH treatment in mice was efficacious in restoring neurogenesis and to counteract this pro-aging phenotype (Zhang et al., 2013).

### Endocrine control and disruptors

Dysfunctions of the hypothalamic neurogenesis, as well as the mutations of the transcription factors controlling hypothalamic development, may led to several endocrine, neurological and psychiatric diseases (OMIM, 2014). These developmental defects usually lead to growth failure and disorders of puberty, which can be either precocious or delayed. Genetic alterations of hypothalamic development include the more common neurohypophiseal diabetes insipidus, as well as central hypogonadism, optic nerve hypoplasia, obesity and Prader-Willi like syndrome, hypothalamic hamartoma, mental retardation and holoprosencephaly that are frequently accompanied by other brain or phenotypic and psychiatric anomalies (Michaud, 2001). Also, hypothalamic insufficiency in adults can be associated to dementia, disturbances in appetite and sleep, as well as hormonal deficiencies.

Noteworthy, hypothalamic neurogenesis could be the target of environmental insults and the so called “endocrine disrupting chemicals”, defined as exogenous substance or mixture that alters function(s) of the endocrine system (Parent et al., 2011), are among the best candidates. The hypothalamic ME, in contrast to other hypothalamic regions, lies outside of the blood-brain barrier, and is thus directly exposed to circulating toxins and pathogens, as well as nutrients that can lead to cellular injury when in oversupply. No clear data are so far available on the effects of the endocrine disruptors on hypothalamic neurogenesis and, in particular, on tanycytes. In a recent report, it has been shown that Zebrafish embryos exposed to a very low dose of either bisphenol A (BPA) or S (BPS), the main analog used in the production of BPA-free products, resulted in an increased hypothalamic neurogenesis with a concomitant hyperactive phenotype (Dawn Kinch et al., 2014).

## Conclusions

The identification of a significant neurogenic activity in postnatal and adult hypothalamus has opened many insights on its physiological and/or pathological significance. However, many aspects still remain to be unveiled, such as the precise rate of hypothalamic neurogenesis in adult human brain. Also, further genetic and functional studies are required to better understand the whole process and how its deregulation might directly contribute to hypothalamus-relevant pathogenesis. Development of *in vitro* hypothalamic NPCs and NSCs systems are also envisaged to clarify the molecular mechanisms regulating their *in vivo* expansion and differentiation into specific hypothalamic neuronal subpopulations and to open to potential future regenerative applications. It can be anticipated that a better understanding of the hypothalamic neurogenesis will not only contribute to explore this phenomenon in humans, but will also be pivotal to improve current pharmacological approaches for many challenging pathologies.

## Conflict of interest statement

The authors declare that the research was conducted in the absence of any commercial or financial relationships that could be construed as a potential conflict of interest.
